# Effect of Co‐Surfactants on Properties and Bactericidal Activity of Cu_2_O and Hybrid Cu_2_O/Ag Particles

**DOI:** 10.1002/open.202300274

**Published:** 2024-03-01

**Authors:** Dam Xuan Thang, Nguyen Thuy Chinh, Nguyen Thi Binh Minh, Trinh Hoang Nghia, Thai Hoang

**Affiliations:** ^1^ Department of Chemical Technology Hanoi University of Industry 298, Cau Dien, Minh Khai, Bac Tu Liem Hanoi 100000 Vietnam; ^2^ Institute for Tropical Technology Vietnam Academy of Science and Technology 18 Hoang Quoc Viet, Cau Giay Hanoi 100000 Vietnam; ^3^ Graduate University of Science and Technology Vietnam Academy of Science and Technology 18 Hoang Quoc Viet, Cau Giay Hanoi 100000 Vietnam; ^4^ Falcuty of Chemistry Hanoi National University of Education 136 Xuan Thuy, Cau Giay Ha Noi 100000 Viet Nam

**Keywords:** copper (I) oxide particles, silver particles, sodium dodecyl sulfate, polyethylene glycol 6000, antibacterial activity, emulsion antibacterial coating

## Abstract

Nanomaterials based on metal oxides, especially Cu_2_O, have received much attention in recent years due to the many unique properties of the surface plasmon resonance they provide. The report presented the co‐precipitation method, a simple preparation method to produce Cu_2_O oxide particles. In addition, to improve the unique antibacterial properties of Cu_2_O, a proposed method is to attach Ag nanoparticles to the surface of Cu_2_O particles. The Cu_2_O and Cu_2_O−Ag particles were synthesized based on redox reactions using ascorbic acid (LAA) as a reducing agent. Moreover, in this experiment, two surfactants, polyethylene glycol 6000 (PEG 6000) and sodium dodecyl sulfate (SDS), were added during the manufacturing process to create particle samples and particle combinations with better properties than the original sample. Changes in the characteristics and properties of particle samples are determined by many different physical and chemical methods such as ultraviolet‐visible spectroscopy (UV‐Vis), infrared spectroscopy (IR), noise X‐ray radiation (XRD), scanning electron microscope (SEM), dynamic light scattering (DLS), energy dispersive X‐ray spectroscopy (EDS), and transmission electron microscopy (TEM). Finally, the activity against bacteria, including *E. coli* and *S. aureus*, was also tested using the agar well diffusion method to determine the zone of inhibition. The results improved the particle size value, which decreased by half to 200 nm when two additional surfactants, PEG and SDS, were added. In addition, the antibacterial ability has also been shown to increase significantly when the diameter of the bacterial inhibition zone increased significantly, reaching values of 20 mm (Cu_2_O/Ag/SDS) and 32 mm (Cu_2_O/Ag/PEG) for the *E. coli* bacterial strain. The initial test sample was only about 14 mm in size. The S. aureus bacterial strain also had a similar improvement trend after adding Ag to the Cu_2_O surface with the appearance of two surfactants, SDS and PEG. The inhibition zone diameter values reached the optimal value at 36 mm in the Cu_2_O/Ag/PEG particle combination sample compared to only the initial 26 mm in the Cu_2_O particle sample. Finally, the particle samples are added to the acrylic emulsion paint film to evaluate the changes. Positive results were obtained, such as improvement in adhesion (1.22 MPa), relative hardness (240/425), and sand drop resistance (100 L/mil) in the Cu_2_O/Ag/PEG particle combination sample, which showed the correctness and accuracy of the research.

## Graphical abstract

This study focuses on a green synthesis of Cu_2_O and Cu_2_O−Ag particles using ascorbic acid (LAA) as a reducing agent in the presence of two surfactants, polyethylene glycol 6000 and sodium dodecyl sulfate. The hybrid materials exhibit strongly antibacterial activity and are potential for the application in the acrylic emulsion coating.

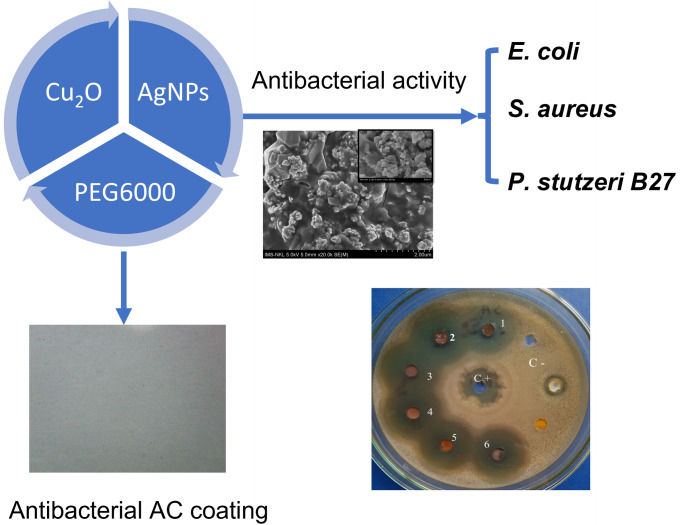

## 1 Introduction

The environment is changing and becoming worse, with urgent problems such as the bacterial pollution and corrosion that was caused by microorganisms. These issues are genuinely instant for the world as each year because of huge losses (approximately tens of millions of dollars annually). In order to handle these outstanding problems, Cu_2_O oxide material immediately attracted attention when it had semiconductor energy of 4.8 eV, showing excellent antibacterial ability. Along with that, the production cost for Cu_2_O is relatively cheap, so it is given priority for research and widespread application in disinfection and antibacterial.[^1^, ^4^] In addition, the antibacterial activity of Cu_2_O is highly appreciated when in the octahedral structure, the bactericidal activity in the environment was determined to reach 98 % and was reported by Wu *et al.*.^[5]^ Furthermore, the inhibition zone of Cu_2_O against *Escherichia coli* (*E. coli*) and *Staphylococcus aureus* (*S. aureus*) strains was shown by Kodasi *et al*. 18 mm and 20 mm, respectively.^[6]^ However, the Cu_2_O has a considerable disadvantage as it is an unstable compound that quickly decomposes or oxidizes under the influence of oxygen, water, light, etc., limiting its long‐term antibacterial activity.[^7^, ^8^] To prevent that, Cai *et al*. used tannic acid (TA) to fabricate a hybrid material (Cu_2_O‐TA) to create a material structure that maintains the chemical stability of Cu_2_O. However, when combined with TA, due to TA′s ability to decompose in marine conditions, the application of Cu_2_O‐TA in the marine field is limited.^[9]^ Therefore, to improve the stability and long‐term antibacterial effect of Cu_2_O, the formation of Cu_2_O/Ag heterostructure is of primary concern. Because silver nanoparticles have antibacterial solid properties with excellent broad spectrum, high durability, and safety, attention is paid to intentionally creating a heterostructure between Cu_2_O and Ag. The photo corrosion phenomenon under light irradiation of Cu_2_O can also be reduced thanks to the heterostructure that prevents photochemical electrons and holes from moving quickly out of Cu_2_O. In addition, the diffusion of Cu^2+^ ions, which are oxidation products from Cu_2_O, into water may be slower due to the distribution of Ag nanoparticles on the surface of Cu_2_O particles. It helps Cu^2+^ ions to maintain the structure of Cu_2_O−Ag as well as prolong the release and long‐term existence of Cu^2+^ ions. These ions on Cu_2_O−Ag also contribute to inhibiting pathogenic bacteria.[^10^, ^11^, ^12^]

Nevertheless, the next problem that scientists pay attention to is when the ability to disperse and dissolve in the water environment of Cu_2_O or even the Cu_2_O−Ag particle combination could be better, leading to its applicability or feasibility. The ability to remove bacteria in some environments is quite limited. Scientists immediately noticed the idea of adding surfactants during manufacturing to improve these properties. In there, sodium dodecyl sulfate (SDS) and polyethylene glycol (PEG) were known co‐surfactants frequently used as additives in the granulation process to eliminate the possibility of aggregation between the particles, thereby decreasing particle size. However, the use of additional PEG or SDS in the synthesis of Cu_2_O particles had so far not been paid much attention, so in this work, the research team conducted a survey and evaluated the possibility of these surfactants in the synthesis process of Cu_2_O and Cu_2_O/Ag particles.[^14^, ^15^, ^16^, ^17^] Notably, these samples, after being fabricated successfully, were characterized by ultraviolet‐visible (UV‐Vis) spectroscopy, Fourier transforms infrared (FTIR) spectroscopy, and inductively coupled plasma mass spectroscopy (ICP‐MS), field emission scanning electron microscopy (FESEM, X‐ray diffraction (XRD) analysis, and dynamic light scattering (DLS) analysis. These samples′ antibacterial activities and MIC value against bacterial strains, including *S. aureus* ‐ a Gram‐positive bacterium, and *E. coli* ‐ a Gram‐negative bacterium, were also evaluated. In particular, it is the first time to consider the bactericidal activity of Cu_2_O and Cu_2_O−Ag against *P. stutzeri* B27 ‐ a Gram‐negative bacterium isolated from marine. Because of this, the requirement to produce coatings or paints that serve many human purposes is increasingly receiving attention. Especially for paint or coating samples, in addition to properties such as stable color quality, color durability, and good coverage, another beautiful property is antibacterial and anti‐fungal properties. Many paint models, such as Kova, TOA Nano Clean, and Dulux Easy Clean, have been successfully researched and applied worldwide. These are all paint models made from acrylic‐based plastic supplemented with antibacterial additives. However, few paint samples use acrylic‐based resin and add Cu_2_O−Ag particle combination with excellent antibacterial properties. Therefore, applying Cu_2_O and Cu_2_O−Ag particles as antibacterial additives for acrylic emulsion coatings to form bactericidal coatings has been tested and discussed.[^14^, ^16^]

## Experimental

### Materials

Silver nitrate (AgNO_3_ >99.7 %), CuSO_4_.5H_2_O (99 %), NaOH (99.5 %), polyethylene glycol 6000 (PEG 6000), sodium dodecyl sulfate (SDS), and L‐ascorbic acid (LAA) were provided by Aladdin Reagent Co., Ltd. (China). Acetone (99.5 %) was a commercial product from Duc Giang Chemical Co. (Vietnam). Bacterial strains (ATCC, Manassas, USA) include *Staphylococcus aureus* (ATCC 6538) – a Gram‐positive bacterium, and *Escherichia coli* (ATCC 8739) ‐ a Gram‐negative bacterium. Marine bacterium ‐ *Pseudononas stutzeri* B27 ‐ a Gram‐negative bacterium was isolated from Phu Quoc Sea, Kien Giang Province (Vietnam). Tryptic soy agar (TSA) was provided by Merck Co. (Germany). Acrylic emulsion resin (AC, commercial name of Plextol R4152) was purchased from Synthomer, USA.

### Synthesis of Cu_2_O particles with or without using co‐surfactants PEG, SDS

The synthesis of Cu_2_O particles was carried out following the procedure reported by Dang Xuan Du and Pham Thi Giang Anh^[18]^ with a bit of change.

Solution A was created in the first step by thoroughly dissolving 2.5 g of CuSO_4_.5H_2_O with or without using 0.15 g PEG or SDS (ratio 1 : 10 with the mass of Cu_2_O particles generated) in 50 mL of the distilled water with stirring magnetically at 400 rpm and room temperature (25 °C ‐ 30 °C). Besides, 0.16 g of NaOH was thoroughly dissolved in 30 mL of the distilled water using a magnetic stirrer to produce solution B. The next step was gradually adding solution B to solution A and stirring the mixture for 30 minutes to form Cu(OH)_2_. At the same time, solution D was created by thoroughly dissolving 0.88 g of LAA (molar ratio of LAA/CuSO_4_.5H_2_O of 1 : 2) in 30 mL of distilled water. Then, the solution D was added dropwise to solution C under a high‐speed ultrasonic stirrer running for 20 minutes at 4000 rpm. The reaction was then finished after 1 hour. The formed mixture will then be settled, filtered, and centrifuged three to four times with acetone solvent to remove residues. The solid part was then collected and dried in an oven at 60 °C within 5 hours to produce Cu_2_O, Cu_2_O/SDS, and Cu_2_O/PEG particles.

### Synthesis of Cu_2_O/Ag particles with or without using co‐surfactants PEG, SDS

The process of fabricating Cu_2_O/Ag particles using two surfactants, SDS and PEG, was similar to synthesizing Cu_2_O particles as mentioned above. However, the LAA weight was 1.76 g to retain an amount of residue LAA after forming Cu_2_O particles. The LAA residue reduces Ag(I) ions to Ag nanoparticles. Another difference in the procedure for the synthesis of Cu_2_O/Ag particles is that after Cu_2_O particles were formed after 1 hour of reaction, the AgNO_3_ solution continued to be dropped into the mixture under the influence of a high‐speed ultrasonic vibration device (4000 rpm). From there, the Ag particles were gradually formed and adhered to the surface of previously synthesized Cu_2_O particles. After the reaction, the solution was filtered, washed with acetone, dried to obtain Cu_2_O/Ag, Cu_2_O/Ag/SDS, and Cu_2_O/Ag/PEG. The composition and abbreviations of their particles are presented in Table 1.

**Table 1:** Composition and abbreviations of Cu_2_O/Ag, Cu_2_O/Ag/SDS, Cu_2_O/Ag/PEG particles.

Symbols of samples	Abbreviations of samples	CuSO_4_.5H_2_O (g)	LAA (g)	SDS (g)	PEG (g)	AgNO_3_ (g)
Cu_2_O	Cu_2_O	2.5	0.88	0	0	0
Cu_2_O/SDS	Cu_2_O/S	2.5	0.88	0.15	0	0
Cu_2_O/PEG	Cu_2_O/P	2.5	0.88	0	0.15	0
Cu_2_O/Ag	Cu_2_O/Ag	2.5	1.76	0	0	0.17
Cu_2_O/Ag/SDS	Cu_2_O/Ag/S	2.5	1.76	0.15	0	0.17
Cu_2_O/Ag/PEG	Cu_2_O/Ag/P	2.5	1.76	0	0.15	0.17

### Preparation of AC coatings containing Cu_2_O, Cu_2_O/Ag particles

The preparation process of emulsion AC coatings would only use the original Cu_2_O, Cu_2_O/Ag particles prepared by the reducing agent of LAA and one of 2 co‐surfactants (PEG) because the investigated properties of coatings contained Cu_2_O/PEG, Cu_2_O/Ag/PEG were better than those of the coatings contained Cu_2_O/SDS, Cu_2_O/Ag/SDS (see subsections 3.1‐3.4). The preparation process of AC‐based coatings includes the following steps:

- 0.02 g of each particle sample was precisely measured into glass vials.
- 0.6 g of distilled water was added to these vials and combined with ultrasonic vibration and magnetic stirring for one hour to obtain a suspension of particles.
- 2 g of AC was added to the suspension, and the mixture was then ultrasonicated and magnetically stirred for two hours to form a uniform mixture.
- The mixture was applied on a seven by 10 cm glass sheet using a film applicator (Erichsen) with a wet thickness of 120 μm.
- These glasses were placed at ambient temperature to dry naturally and prevent breaking so they could be used for the following assessments.

AC coating without particles was prepared as a control sample. The composition and symbols of the coatings are shown in Table 2.

**Table 2:** Composition and symbols of AC coatings contained Cu_2_O, Cu_2_O/Ag particles.

No.	Abbreviations of samples	AC (g)	Cu_2_O (g)	Cu_2_O/P (g)	Cu_2_O/Ag (g)	Cu_2_O/Ag/P (g)	Distilled water (g)
1	Cu_2_O/AC	2	0.02	0	0	0	0.6
2	Cu_2_O/P/AC	2	0	0.02	0	0	0.6
3	Cu_2_O/Ag/AC	2	0	0	0.02	0	0.6
4	Cu_2_O/Ag/P/AC	2	0	0	0	0.02	0.6
5	AC	2	0	0	0	0	0.6

### Characterization

UV‐Vis diffuse reflectance spectra (DRS) of the Cu_2_O, Cu_2_O/Ag particles synthesized with or without using co‐surfactants PEG, SDS were performed on a Shimadzu UV‐2450 spectrometer (Japan) in a wavelength range from 400 to 800 nm. FTIR spectra of these particles were recorded using a Nicolet iS10 spectrometer (Thermo Scientific, USA) in the wavenumber range from 4000–400 cm–^1^ with a resolution of 8 cm–^1^ and scans of 16 times. The particles were pelleted with KBr. Their field emission scanning electron microscopy (FESEM) images were taken using an S4800 FESEM (Hitachi, Japan) with a magnification of 1000 to 300,000 times. The size distribution of these particles was measured on an SZ‐100Z2 nanoparticle analyser (Horiba, Japan). XRD patterns of the samples were recorded using a Siemens D5000 (Germany) with the wavelength of CuKa of 0.154 nm, step of 0.03°, and range of 2‐theta of 5–70°. The reaction yield of the synthesis process of Cu_2_O/P and Cu_2_O/Ag/P particles was calculated based on the ICP‐MS method. To do this, after being prepared, 0.01 g of samples was wholly dissolved in 10 mL of nitric acid. Then, the ICP‐MS spectra of these solutions were recorded on a Single Quadrupole ICP‐MS device to determine the concentration of Cu_2_O and Ag in Cu_2_O/P and Cu_2_O/Ag/P particles.[^17^, ^18^, ^19^, ^20^]

Antibacterial activities of samples were evaluated using the diffusion method on an agar well. The particle samples were dispersed in distilled water supported by ultrasonication and added into the agar wells with a volume of 50 mL. The agar dishes were cultured at 4 °C for four hours at 37 °C. After 24 hours of testing, the inhibition zone of the samples was observed and measured. The bacterial strains *S. aureus* (ATCC 6538), *E. coli* (ATCC 8739) and *P. stutzeri* B27. Ampicillin was used as a positive control sample, and reverse osmosis (RO) water was used as a negative control sample. The minimum inhibitory concentration (MIC) value of samples was determined by diluting the sample suspension (100 mg/mL) to 10, 50, 100, 500, and 1000 times corresponding to 10, 2, 1, 0.2, and 0.1 mg/mL to observe the lowest concentration that could inhibit the growth of bacteria in agar wells.

The mechanical properties of AC‐based coatings, including impact strength, abrasion resistance, adhesion, and relative hardness, were also determined. For instance, the abrasion resistance was evaluated by a Falling Sand Abrasion Tester according to ASTM D968‐15, the impact strength was tested by an Erichsen impact tester (model 304), an Elcometer F510‐20s device measured the adhesion, the relative hardness was conducted by Pendulum Damping Tester Model 299/300.

### Statistics analysis

The statistical analysis of this report is calculated based on the conduct of experiments with a repeatability of 3 times. The results obtained will be processed through Origin software to calculate mean values, variance, and error.

## 2 Results and Discussion

### 2.1 UV‐Vis diffuse reflectance spectra of Cu_2_O, Cu_2_O/Ag particles

**Figure 1:**
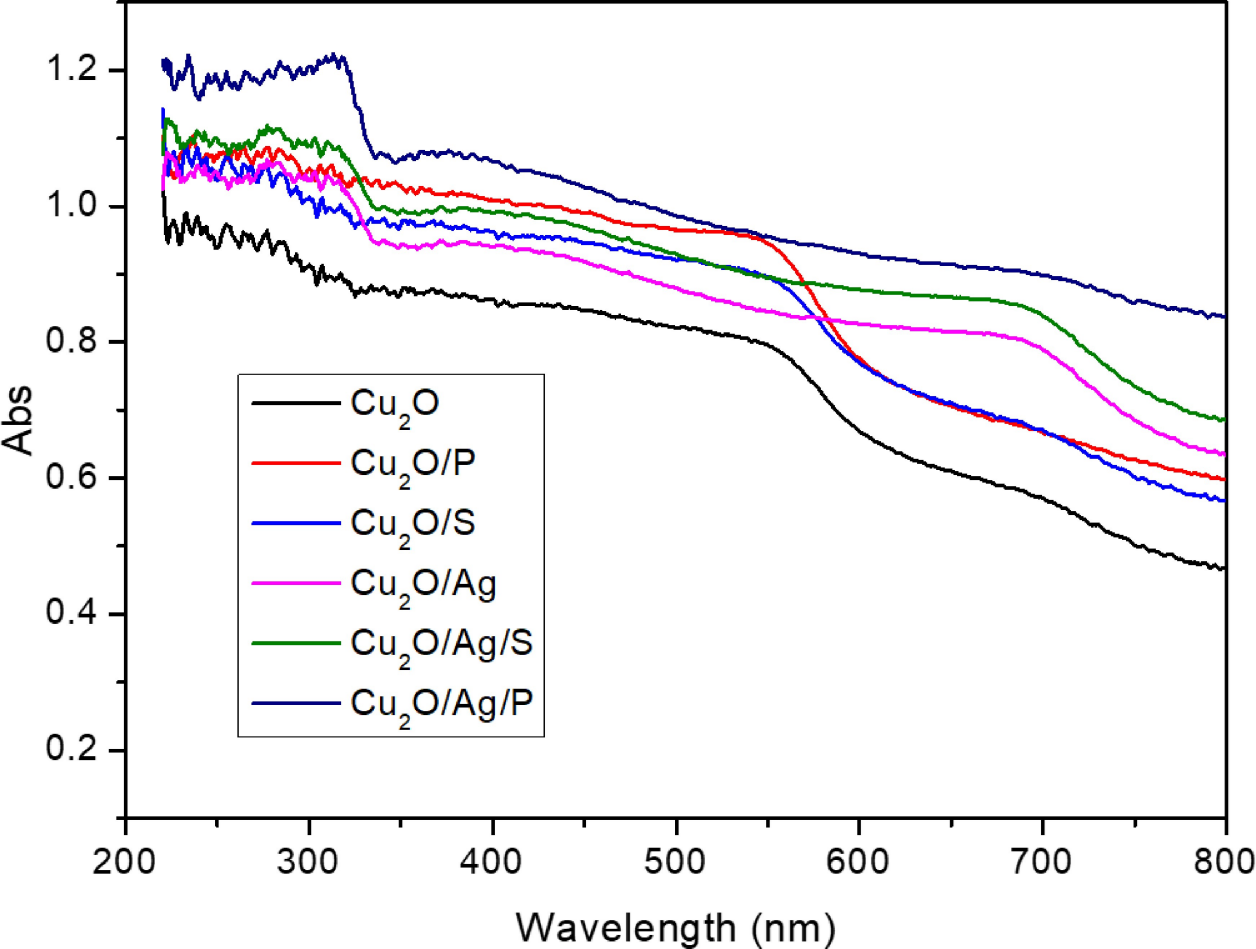
UV‐Vis diffuse reflectance spectra of Cu_2_O, Cu_2_O/Ag particles.

The UV‐Vis diffuse reflectance spectra of Cu_2_O and Cu_2_O/Ag particles are displayed in Figure 1. It was expected to obtain the Cu_2_O‐specific absorption peak at about 550 nm on the visible region characterized by an electron transition from the valence band to the conduction band.^[19]^ However, when SDS and PEG surfactants were added during the synthesis process, it helped Cu_2_O particles avoid clumping together, which may be why the absorption range of Cu_2_O widened and became more apparent. Consequently, the intensity of the absorbance peak of Cu_2_O was also increased and ranged in an order of Cu_2_O/P > Cu_2_O/S > Cu_2_O. The same trend was observed for Cu2O/Ag particles with surfactants. The absorbance peak of Cu_2_O at about 550 nm in the UV‐Vis diffuse reflectance spectra of Cu_2_O/Ag is similar to that of the Cu_2_O, while a broad absorbance peak at approximately 690 nm attributed to the heterostructure of the Cu_2_O/Ag.[^19^, ^20^] When using surfactants, this peak becomes more sharply.

**Figure 2:**
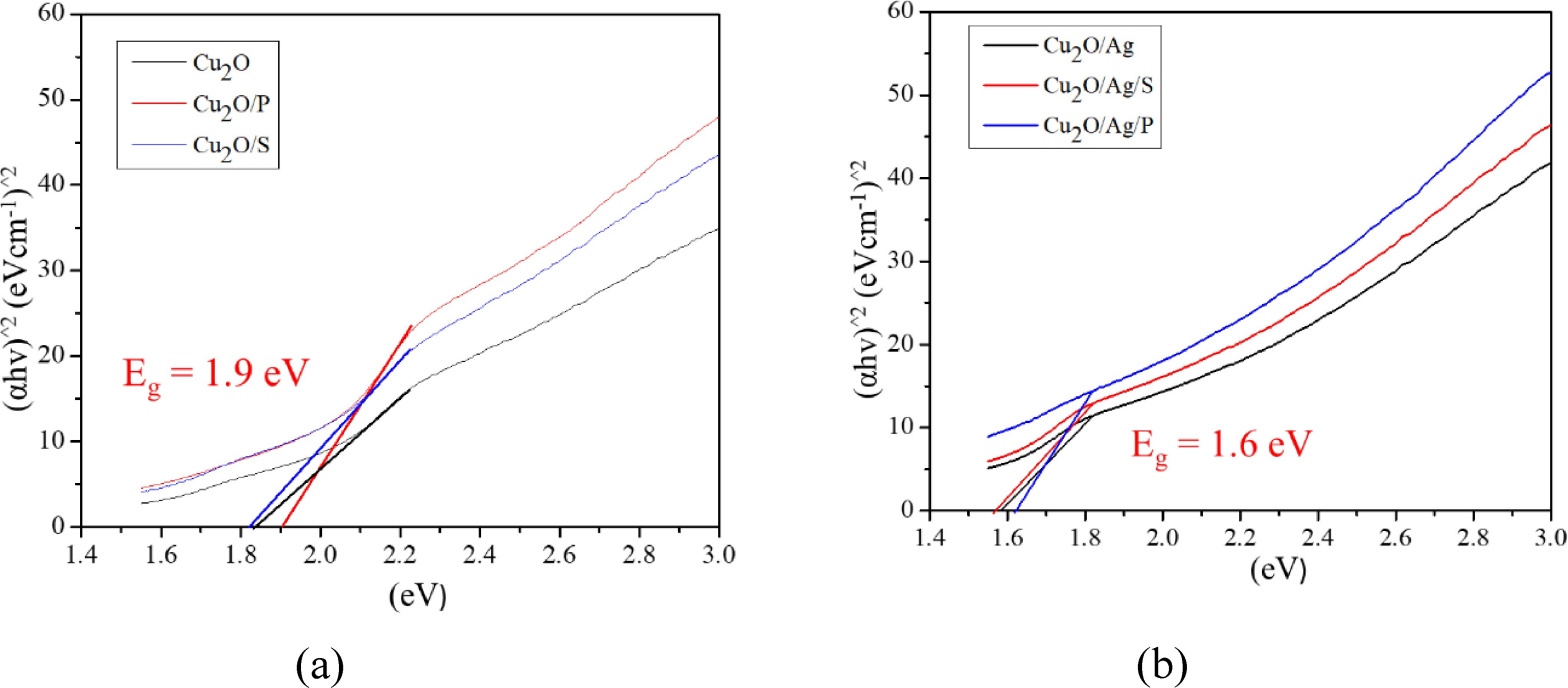
Tauc's plot of Cu_2_O (a) and Cu_2_O/Ag particles (b).

The obtained UV‐Vis diffuse reflectance results were used to calculate the band gap energy of the fabricated particles based on the Tauc′ plot. The results are presented in Figure 2. The band gap energy values of Cu_2_O, Cu_2_O/P, and Cu_2_O/S were about 1.9 eV, while those of Cu_2_O/Ag, Cu_2_O/Ag/P, and Cu_2_O/Ag/S were about 1.6 eV. It is seen that the combination of Ag nanoparticles with Cu_2_O contributed to the decrease of band gap energy of the Cu_2_O samples, but not too much. It is caused by the Schottky barrier effect and heterostructure.[^18^, ^19^, ^20^] These results were consistent with W. Zhang et al.′s report on the Cu_2_O/Au particles.^[21]^ Finally, the spectral peak at 350 nm was said to be characteristic of the plasmonic surface resonance of Ag nanoparticles with the Cu_2_O particle surface.

### 2.2 Yield of synthesis process for Cu_2_O and Cu_2_O/Ag particles

**Figure 3:**
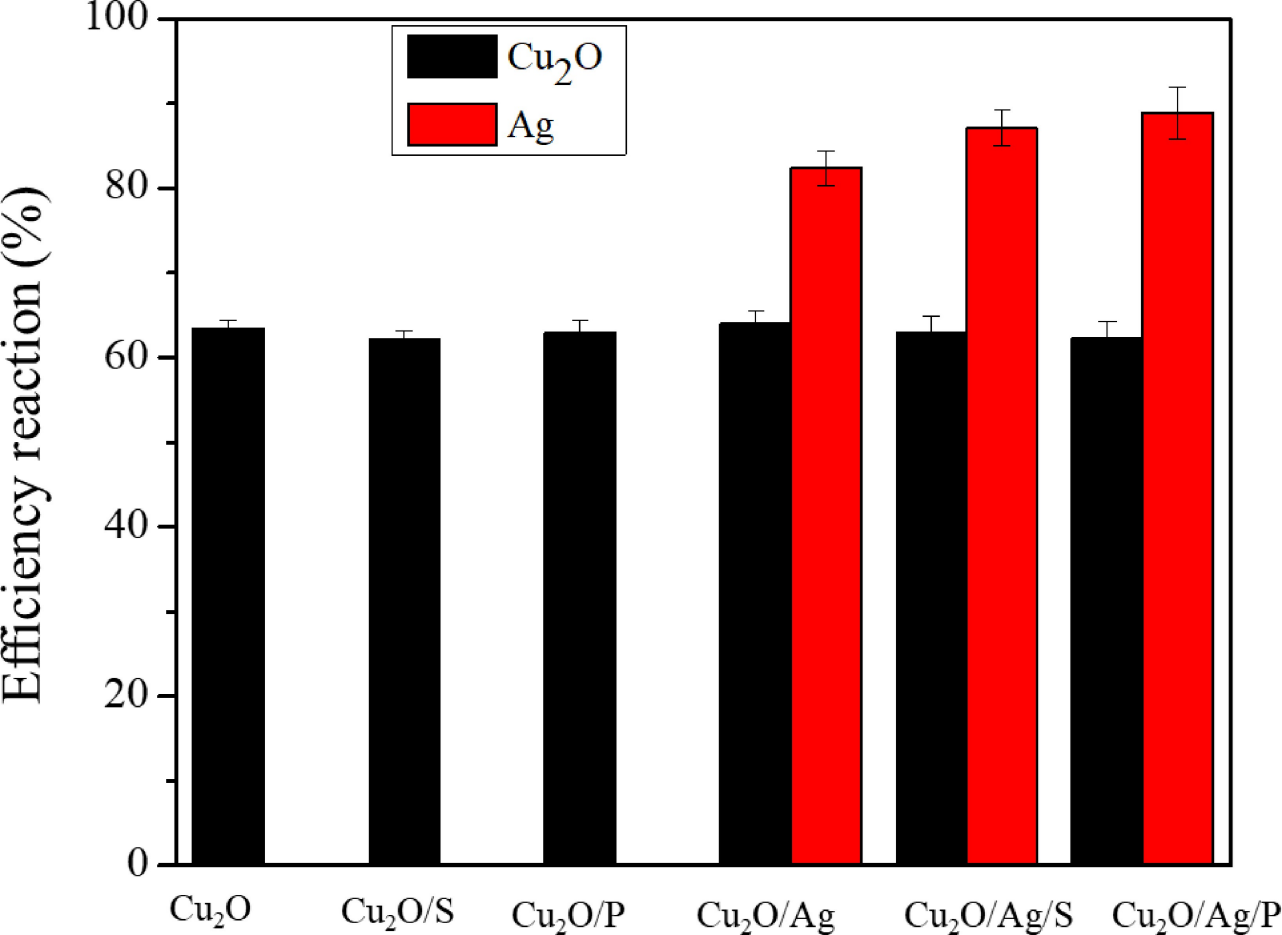
Yield of synthesis process of Cu_2_O, Cu_2_O/Ag particles.

The yield of the synthesis process of Cu_2_O and Cu_2_O/Ag samples can be estimated from the results of the ICP‐MS analysis. In addition, surfactants did not cause a change in the yield of the synthesis process of the Cu_2_O sample. The yield of the synthesis process of Cu _2_O was medium, reaching above 60 %. In particular, the presence of surfactants had a positive effect on the yield of the synthesis process of AgNPs on Cu_2_O. For instance, the yield of the synthesis process of the AgNPs on Cu_2_O reached 88.89 %, 89.47 %, and 88.93 % for the Cu_2_O/Ag, Cu_2_O/Ag/P, and Cu_2_O/Ag/S particles, respectively (Figure 3).

### 2.3 FTIR spectra of Cu_2_O, Cu_2_O/Ag particles

**Figure 4:**
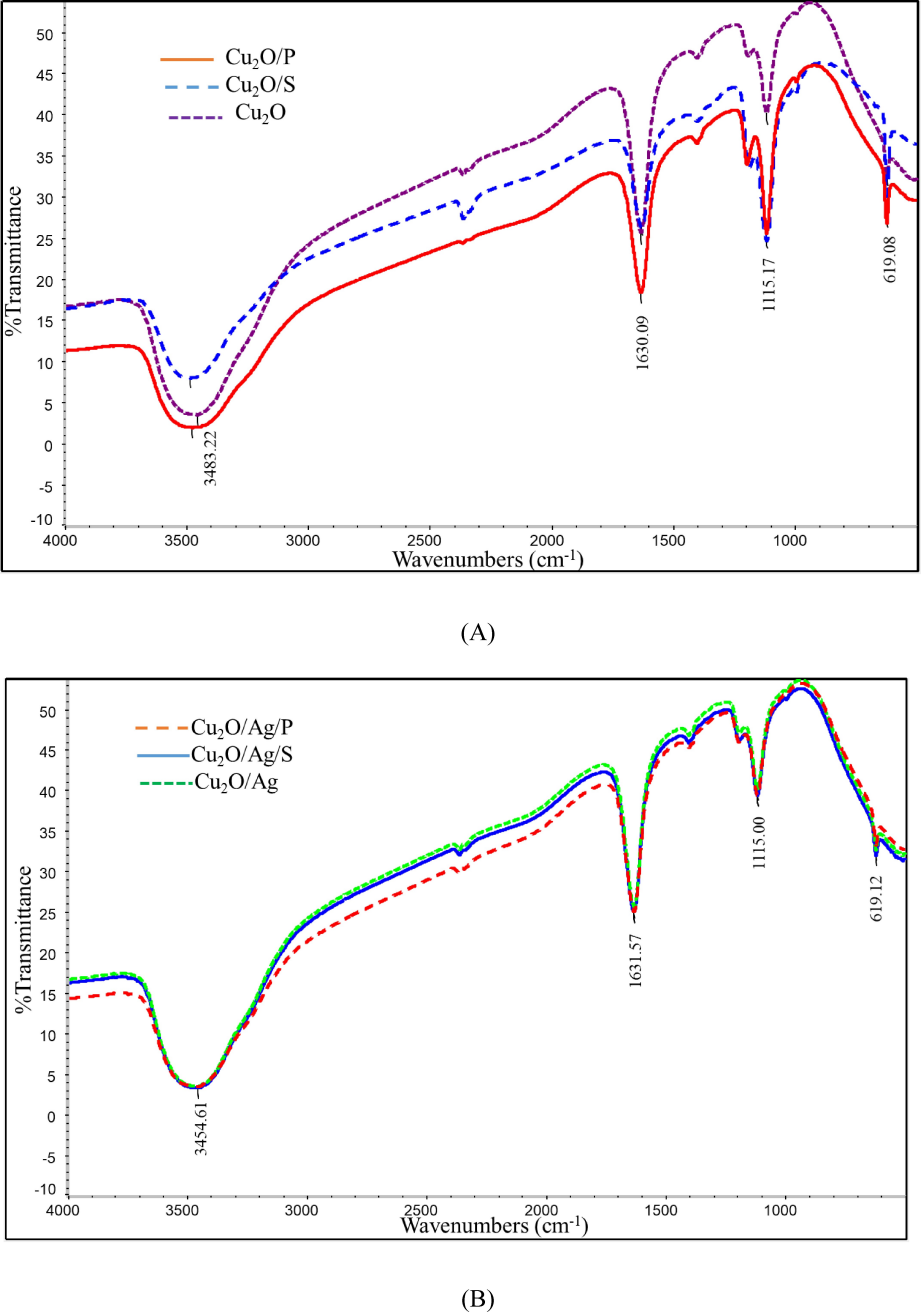
FTIR spectra of Cu_2_O (A), Cu_2_O/Ag (B) particles.

FTIR spectra of Cu_2_O and Cu_2_O/Ag particles are displayed in Figure 4. As observed from the FTIR spectrum of Cu_2_O, the peaks at a wavenumber of 619 cm–^1^ characterized Cu(I)−O.^[22]^ In the presence of surfactants, the change in these vibrations in the FTIR spectra of Cu_2_O/S, Cu_2_O/P, Cu_2_O/Ag, Cu_2_O/Ag/S, Cu_2_O/Ag/P particles samples compared with the Cu_2_O sample was negligible. Interestingly, a new peak at about 1100 cm–^1^ that characterized C−O linkages in the surfactants appeared in the FTIR spectra of Cu_2_O/S, Cu_2_O/P, Cu_2_O/Ag/S, and Cu_2_O/Ag/P samples, suggesting that a tiny part of surfactants remained in the structure of samples. The primary role of surfactants in the Cu_2_O synthesis process is to prevent agglomeration of Cu_2_O to form more giant clusters. Although the surfactants were washed when the reaction finished, one part was attached deeply inside the structure of the particles. Moreover, with the Ag nanoparticles (AgNPs) successfully attached to the surface of the Cu_2_O particles, the characteristic peak representing the Cu(I) ‐ O bond at 619 cm–^1^ had a marked decrease, suggesting that the Cu_2_O particles had participated in the formation of heterostructure with AgNPs.[^21^, ^22^] The presence of LAA was also characterized by the C double bond C at a value of about 1630 cm–^1^. Finally, the peaks observed at 3481 cm–^1^ correspond to the hydroxyl groups.[^20^, ^22^]

### 2.4 Size distribution of Cu_2_O, Cu_2_O/Ag particles

**Figure 5:**
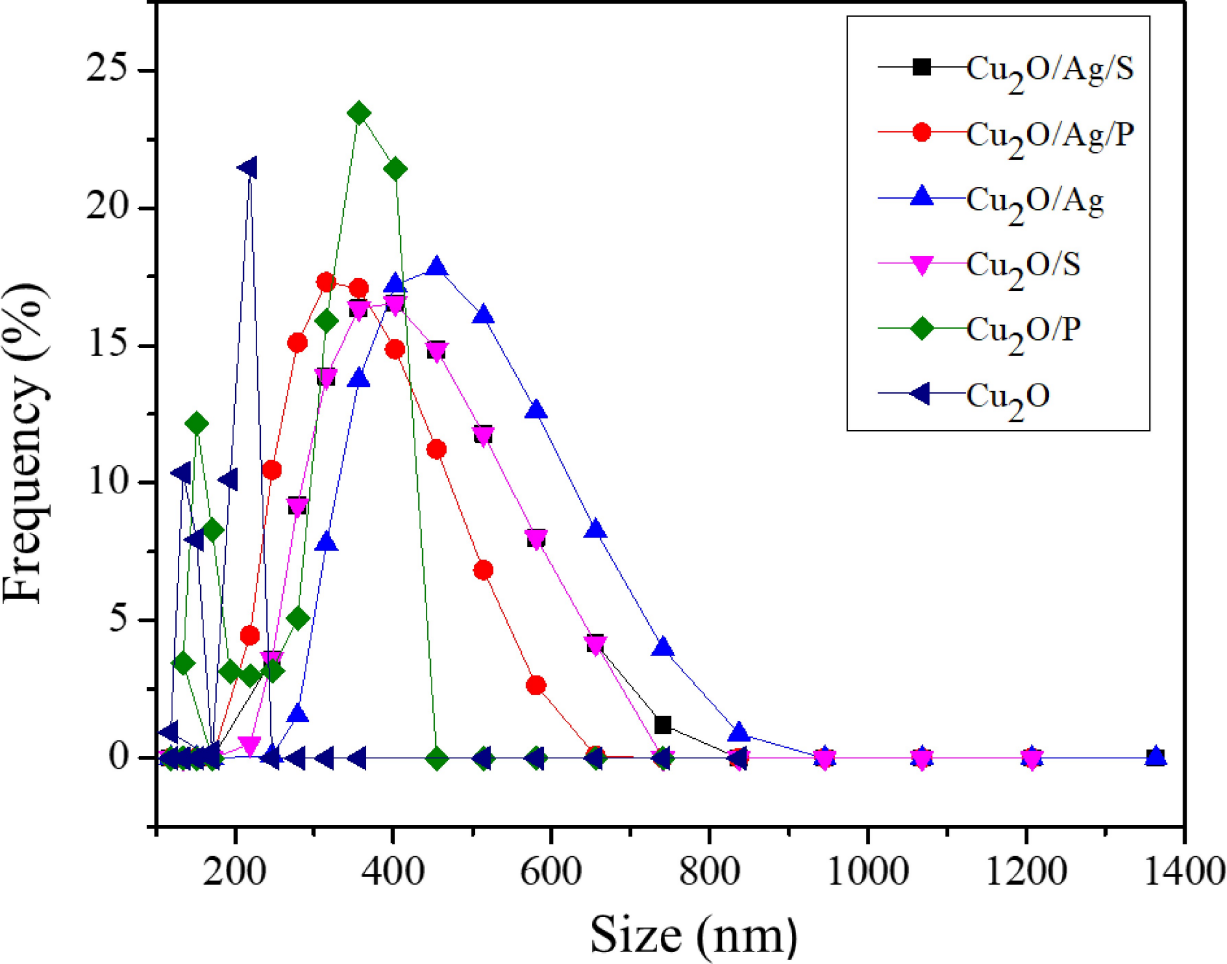
Size distribution diagrams of Cu_2_O, Cu_2_O/Ag particles.

Figure 5 illustrates size distribution diagrams of Cu_2_O and Cu_2_O/Ag particles. The size distribution, average size, peak mean, and polydispersity index (PI) of Cu_2_O and Cu_2_O/Ag particle samples are presented in Table 3. It was clear that the average particle size and peak size values of Cu_2_O/S and Cu_2_O/P particles tended to decrease as compared to those of the Cu_2_O particles. For example, the average size values of Cu_2_O, Cu_2_O/S, and Cu_2_O/P particles were 418.5 nm, 328.9 nm, and 298.6 nm, respectively. In addition, the PI of Cu_2_O and Cu_2_O/Ag particles also indicated that these particle samples were polydispersity, and the addition of SDS and PEG would significantly enhance the water dispersion and stability of the fabricated particle samples. As observed in the data in Table 3, the average particle size of Cu_2_O/P particles was much lower than that of the Cu_2_O/S and Cu_2_O particles. It can be explained by the structure of PEG containing a lot of OH groups, so when it was attached to the Cu_2_O/P particles, it would help to limit particles agglomerated, leading to contributing better dispersion of the particles in water, thus the PI value of Cu_2_O/P particles was the lowest.[^23^, ^24^, ^25^]

**Table 3:** Average size, peak size, and polydispersity index (PI) of Cu_2_O, and Cu_2_O/Ag particles.

Sample	Z‐Average (nm)	Peak size (mean ± SD)	PI
Cu_2_O/Ag/S	497.1±0.2	430.6±17.2	0.491
Cu_2_O/Ag/P	476.5±0.6	331.0±11.21	0.416
Cu_2_O/Ag	523.8±0.5	456.2±17.1	0.521
Cu_2_O/S	328.9±0.8	228.2±61.9	0.497
Cu_2_O/P	298.6±0.2	225.3±60.0	0.466
Cu_2_O	418.5±0.4	327.3±19.4	0.662

When the Cu_2_O particles were combined with AgNPs, the average particle size of Cu_2_O/Ag particles prepared by using surfactants was slightly reduced in the presence of surfactant. This may be because of a stable heterostructure of the Cu_2_O/Ag particles that was difficult to break into smaller particles.

When the Cu_2_O particles were combined with AgNPs, the average particle size of Cu_2_O/Ag particles prepared using surfactants was slightly reduced in the presence of surfactant. This may be because of a stable heterostructure of the Cu_2_O/Ag particles that was difficult to break into smaller particles.

**Figure 6:**
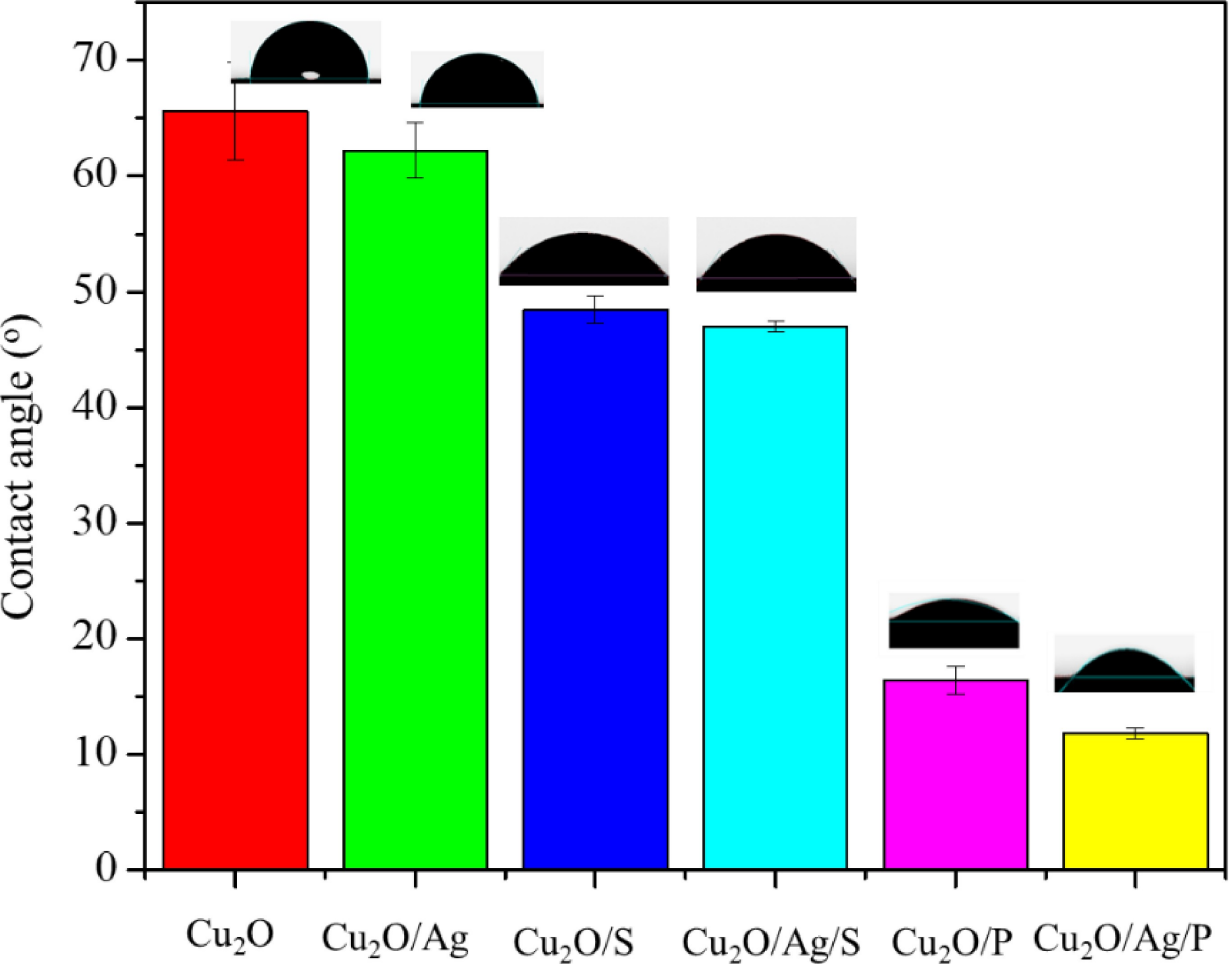
Contact angle of Cu_2_O and Cu_2_O/Ag particles.

Moreover, the average particle size value of Cu_2_O and Cu_2_O/Ag samples was decreased when adding two surfactants, SDS and PEG, to the synthesis process of Cu_2_O and Cu_2_O/Ag particles could also be explained by their contact angle value determined and shown in Figure 6. It was easy to see a sharp decrease in the contact angle value between the Cu_2_O, Cu_2_O/Ag particles and Cu_2_O/S, Cu_2_O/P, Cu_2_O/Ag/S, and Cu_2_O/Ag/P particles. The higher the contact angle value, the worse the dispersion and water solubility of the particles, so the addition of PEG in the synthesis process caused the contact angle value to decrease sharply from about 70° to approximately 16°, showing that the dispersion of particles in the water was greatly enhanced. Since then, the agglomeration of particles has been limited, so the determined average size values of particles were also significantly reduced.[^23^, ^26^, ^27^] These results showed that the decrease in the contact angle of the sample was caused by two surfactants, PEG and SDS, which act with the penetration effect rather than the wetting effect.

### 2.5 Morphology of Cu_2_O, Cu_2_O/Ag particles

**Figure 7:**
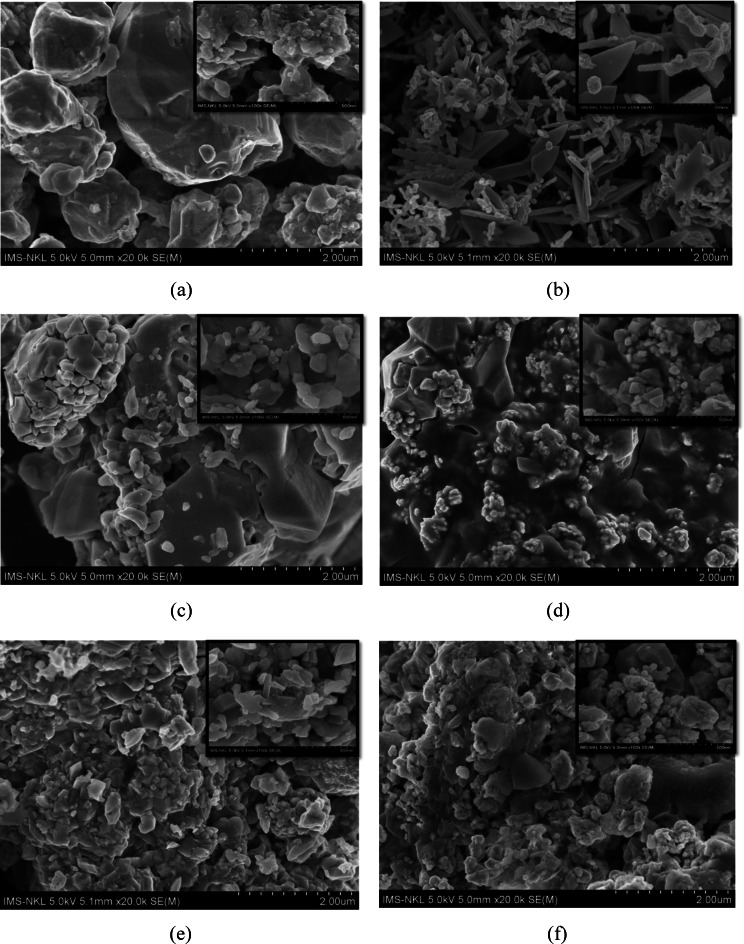
SEM images of fabricated particles: Cu_2_O (a), Cu_2_O/Ag (b), Cu_2_O/P (c), Cu_2_O/Ag/P (d), Cu_2_O/S (e), Cu_2_O/Ag/S (f).

The SEM images of Cu_2_O and Cu_2_O/Ag particle synthesis with or without using co‐surfactants of PEG and SDS are displayed in Figure 7. From Figure 7a, as viewed from the SEM images at a magnification of 100k times, the Cu_2_O particle samples had a smooth surface with a cubic structure characterized for Cu_2_O crystals with a particle size of ca. 100–250 nm, and these particles were agglomerated with each other. Consequently, adding two co‐surfactants, PEG and SDS, to the synthesis process of Cu_2_O particles reduced the agglomeration of Cu_2_O particles significantly (Figure 7 a, c, e). It was characterized by the properties of two surfactants, SDS and PEG, which helped to prevent the Cu_2_O particles from sticking together.[^24^, ^25^, ^26^] Interestingly, as observed in Figures 7b, 7d, and 7 f, the AgNPs in bright spherical form were attached to the surface of Cu_2_O particles through a chemical bond of Cu−Ag. The AgNPs were most clearly observed in particle samples with PEG surfactant added, even at a slight magnification of only 20k (Figure 7d). Thus, it took time to observe the spherical structure of AgNPs on Cu_2_O/Ag and Cu_2_O/Ag/S particles in Figures 7b and 7 f. The AgNPs were seen more clearly in SEM images with higher magnification at 100k for these samples; they were also easy to agglomerate into large clusters.[^27^, ^28^] The presence of PEG co‐surfactant not only helps to decrease the aggregation of Cu_2_O particles but also helps to improve the dispersion of AgNPs on the surface of Cu_2_O particles.

**Figure 8:**
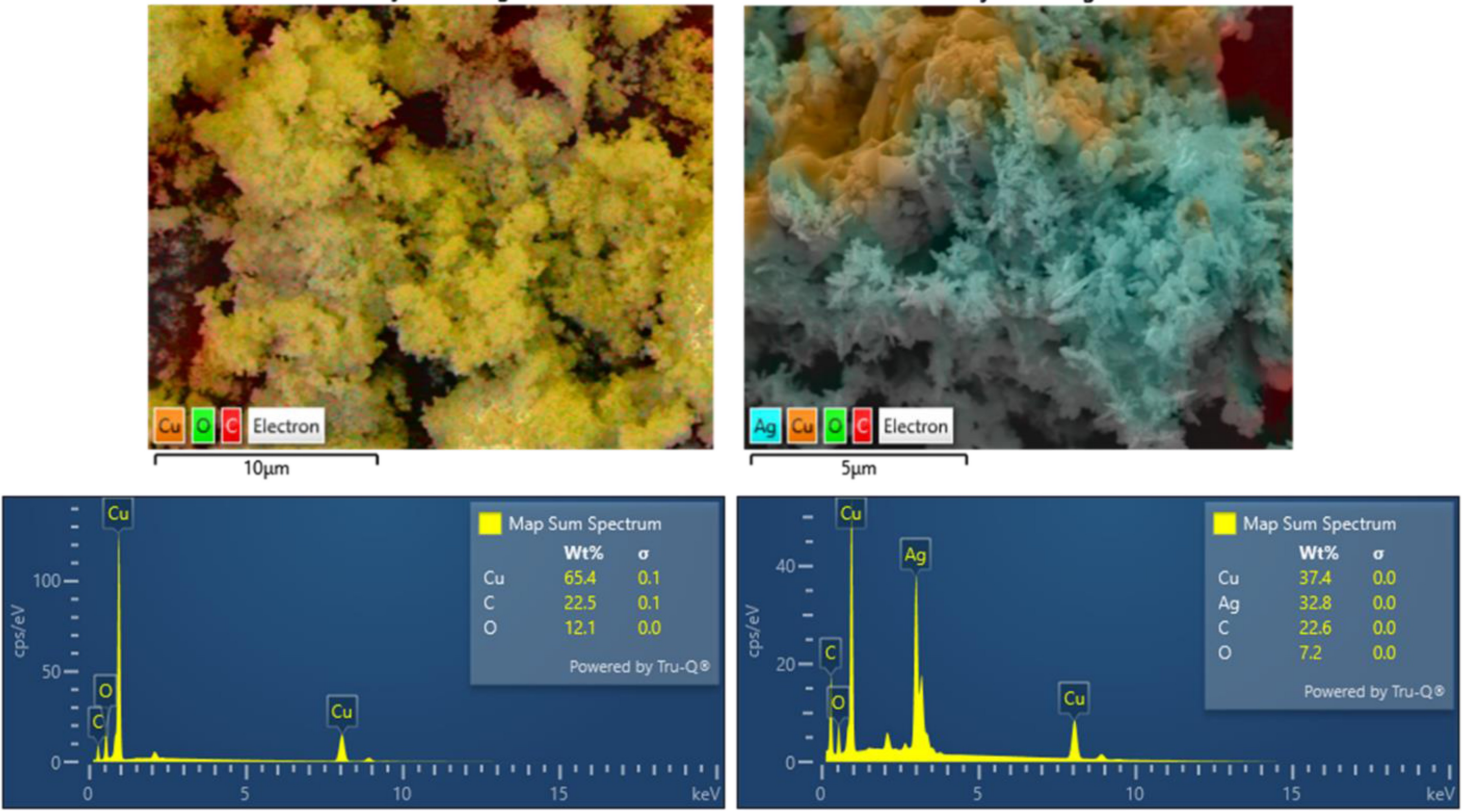
EDS mapping and EDX analysis results of samples Cu_2_O and Cu_2_O/Ag.

**Figure 9:**
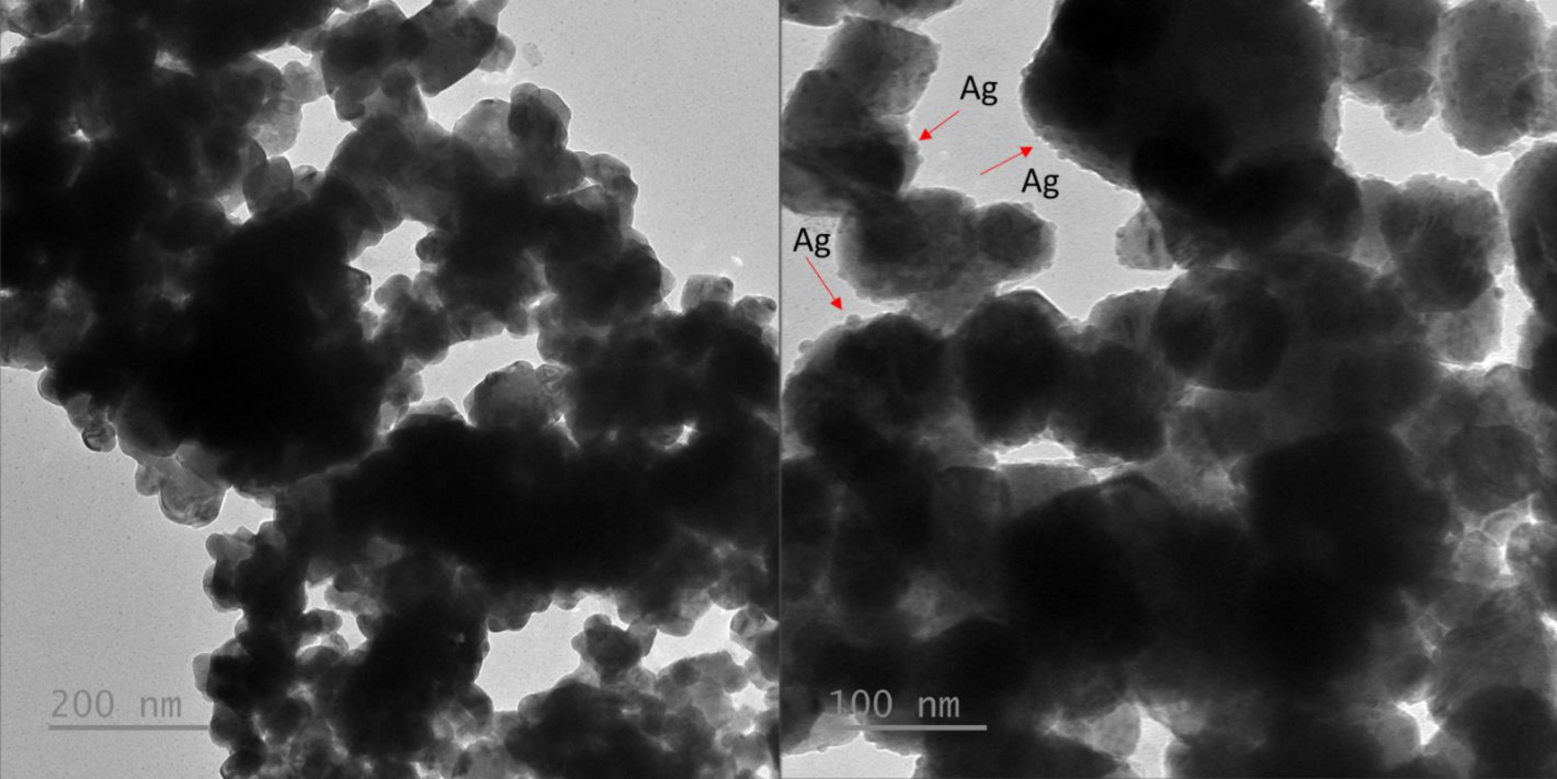
TEM images of fabricated samples of Cu_2_O and Cu_2_O/Ag particles.

The analysis results based on EDS mapping, EDX phase composition, and TEM images of Cu_2_O/P and Cu_2_O/Ag/P samples are also reported in Figures 8 and 9. The EDX phase composition and EDS mapping demonstrate the presence of Cu in Cu_2_O/P sample while Cu and Ag in the Cu_2_O/Ag/P sample's composition. For the Cu_2_O/P sample, the content of Cu element is the largest, then C and O. The existence of C on the surface of Cu_2_O/P sample due to the residue of PEG in the sample. For the Cu_2_O/Ag/P, it can be seen the distribution of Cu and Ag on the surface of sample. Ag element presented in the surface of sample with a position close to Cu and another only Ag element. This can suggest that Ag formed in the sample in two forms, a heterogenous structure Cu−Ag and AgNPs. However, Ag nanoparticles tend to agglomerate to each other. This can be reflected more clearly through the TEM analysis. From TEM images of Cu_2_O/P and Cu_2_O/Ag/P in Figure 9, after the Ag NPs attachment process, the TEM images also clearly show the structural change of Cu_2_O when seeing the heterostructure formed between Cu_2_O−Ag and the Ag nanoparticles on the surface of Cu_2_O.

### 2.6 XRD patterns of Cu_2_O, and Cu_2_O/Ag particles

**Figure 10:**
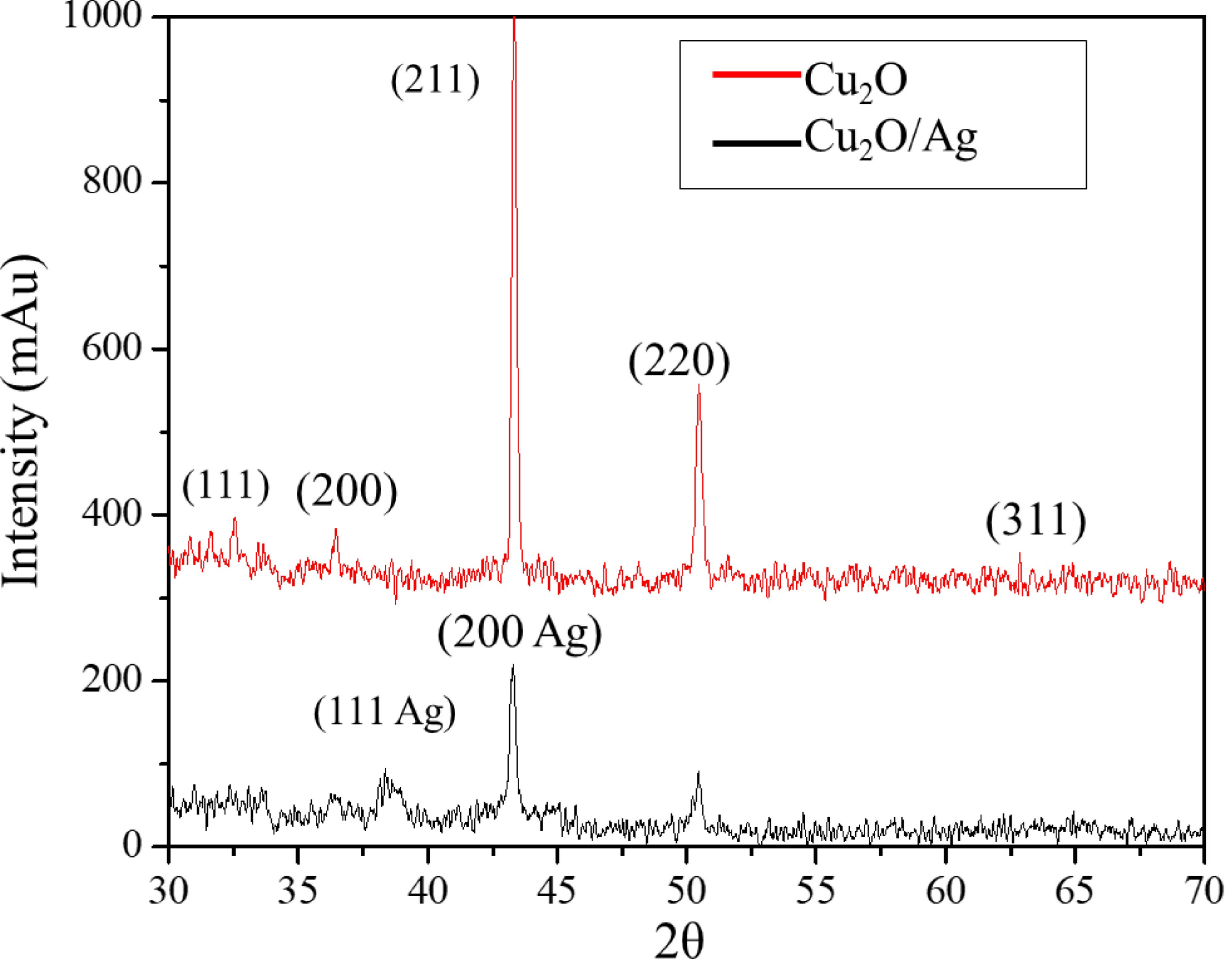
XRD patterns of Cu_2_O and Cu_2_O/Ag particles.

The XRD method has investigated the composition and phase purity of Cu_2_O and Cu_2_O/Ag particles. As the XRD patterns of Cu_2_O and Cu_2_O/Ag particles displayed in Figure 10, the diffraction peaks of Cu_2_O particles at 29.65°, 36.52°, 42.42°, and 61.54° were assigned to the (1 1 0), (1 1 1), (2 0 0), (2 2 0), (3 1 0), (3 1 1) and crystal planes of Cu_2_O (JCPDS card No. 05‐0667) phase.[^26^, ^29^] No other peaks appeared between the diffraction peaks, indicating that the prepared Cu_2_O particles had high purity. Moreover, based on the values (h, k, l) in the lattice corresponding to theta angles determined in this experiment, the characteristic α for the formed Cu_2_O particles was 4.26. This result agreed with the theory calculation that Cu_2_O particles in the cubic form would have a value α index of 4.27. It could be concluded that the produced Cu_2_O particles had a cubic structure. The XRD pattern of Cu_2_O/Ag particles not only exhibited the diffraction peaks of the Cu_2_O phase but also showed firm diffraction peaks at 38.26°, 44.47° corresponding to (1 1 1), (2 0 0), crystal planes of Ag (JCPDS card no. 87‐0718) phase,^[30]^ indicating that the AgNPs were successfully formed on the surface of Cu_2_O particles.

### 2.7 Antibacterial activity of Cu_2_O and Cu_2_O/Ag particles

**Figure 11:**
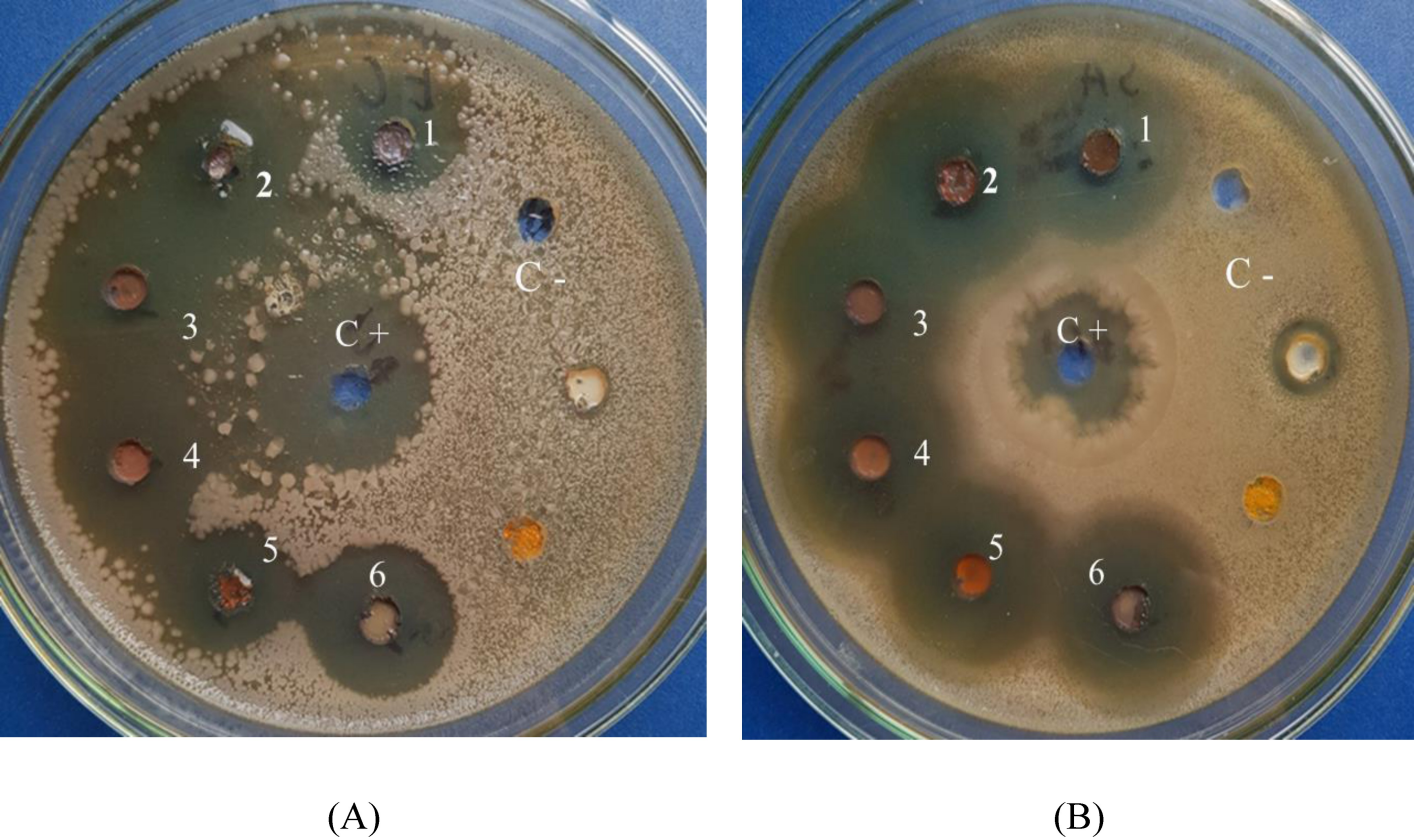
Agar dish images of testing against *E. coli* (A) and *S. aureus* (B) of all investigated samples after 24 h of culturing at 37 °C.

The antibacterial activity of all particle samples was determined by calculating the inhibition zone of the samples. Firstly, 0.01 g of particle samples were weighed with ultrasonic vibration to disperse in 1 ml of distilled water. Then, each sample was tested against bacteria with two strains, *E. coli*, and *S. aureus*, by determining the sterile ring after 24 hours of testing at 37 °C. As observed in Figure 11 and Table 4, the Cu_2_O and Cu_2_O/Ag particles had good resistance to two types of bacteria, *E. coli* and *S. aureus*, through the value of the inhibition zone was over 10 mm. However, a positive change in the diameter of the inhibition zone is a testament to the antibacterial ability of the Cu_2_O and Cu_2_O/Ag samples fabricated by using SDS and PEG surfactants. For example, with the *E. coli* strain, the inhibition zone of Cu_2_O, Cu_2_O/S, and Cu_2_O/P particles was 14 mm, 16 mm, and 18 mm, respectively.

**Table 4:** Inhibition zone (D‐d) of Cu_2_O and Cu_2_O/Ag particles.

No.	Name of sample	Concentration (mg/mL)	Inhibition zone (D‐d) (mm)	
			*E. coli*	*S. aureus*
1	Cu_2_O	100	14±3	26±0
2	Cu_2_O/S	100	16±2	26±0
3	Cu_2_O/P	100	18±3	28±2
4	Cu_2_O/Ag	100	18±3	30±4
5	Cu_2_O/Ag/S	100	20±2	30±2
6	Cu_2_O/Ag/P	100	32±3	36±3
C‐	Negative control	50	0	0
C+	Ampicillin	10	22	34

Moreover, the Cu_2_O/Ag particles had a better antibacterial ability, as shown by the value of the inhibition zone to over 20 mm, and the highest value was 32 mm of Cu_2_O/Ag/P particles. It proved that the attachment of Ag particles to the surface of Cu_2_O particles was utterly practical because the solid and sensitive antibacterial activity of Ag nanoparticles increased the bactericidal ability of Cu_2_O/P and Cu_2_O/Ag/P particles significantly. For instance, in comparison of the inhibition zone for the *S. aureus* strain between the Cu_2_O and Cu_2_O/Ag particles, the inhibition zone was increased from 26 mm to 30 mm. In addition, the Cu_2_O/Ag/S and Cu_2_O/Ag/P particles also had a higher inhibition zone value than the Cu_2_O/Ag particles. This indicated that adding PEG and SDS surfactants to the fabricated process of Cu_2_O and Cu_2_O/Ag particles also enhanced the antibacterial activity of Cu_2_O and Cu_2_O/Ag particles. It could be explained that the addition of two surfactants, PEG and SDS, to the synthesis process of Cu_2_O/S, Cu_2_O/P, Cu_2_O/Ag/S, and Cu_2_O/Ag/P particles contributed to improving the diffusion to the agar of particles thanks to a smaller size to kill the bacteria, making these particles had a better antibacterial activity.[^31^, ^32^, ^33^]

Finally, through all the obtained data, it was found that the sample of Cu_2_O/Ag/P particles had the best antibacterial activity against both bacterial strains *E. coli* and *S. aureus* when the inhibition zone recorded the highest value was 32 mm for *E. coli* and 36 mm for *S. aureus*, respectively. Based on that, the samples of Cu_2_O/P and Cu_2_O/Ag/P particles were conducted to determine the minimum inhibitory concentration and then put into the AC emulsion to make antibacterial activity coatings.

### 2.8 Minimum inhibitory concentration (MIC) for different bacteria by Cu_2_O, Cu_2_O/Ag, Cu_2_O/P, Cu_2_O/Ag/P particles

**Figure 12:**
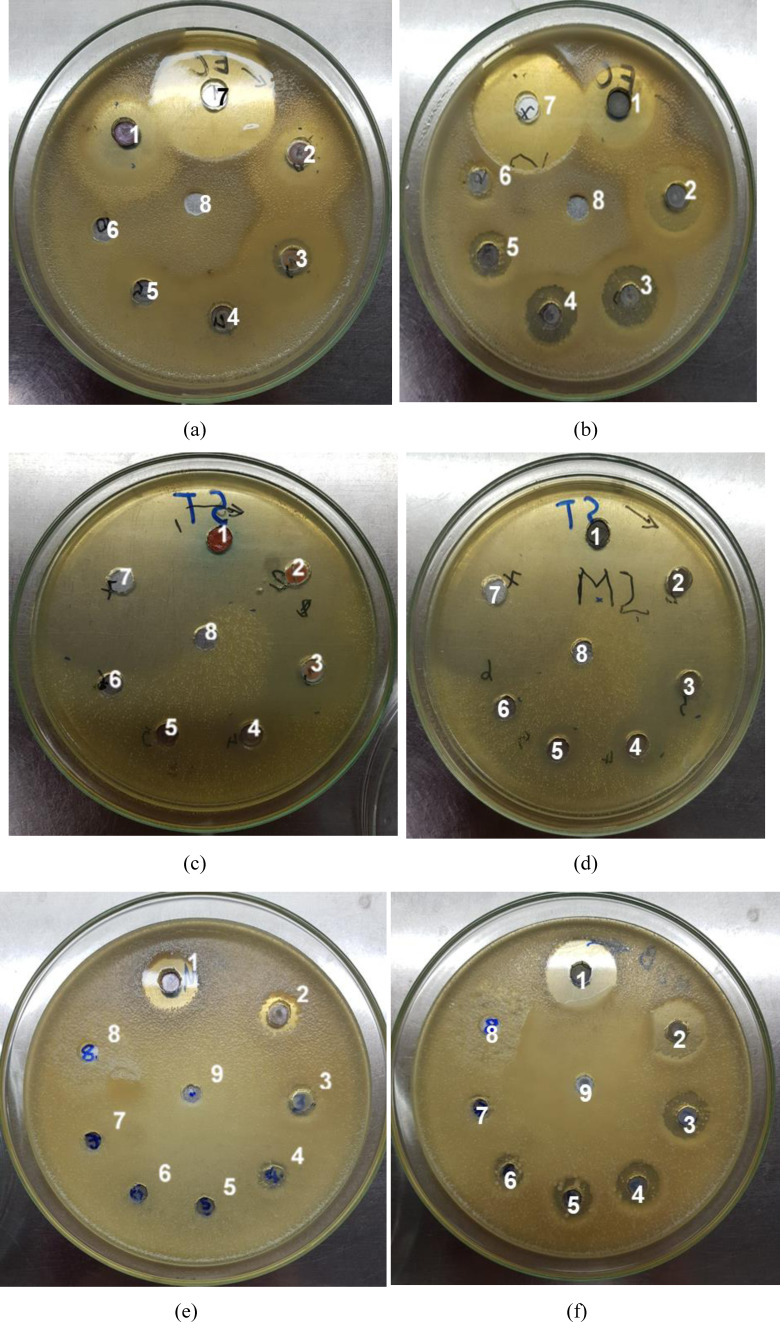
Agar dish images of testing against *E. coli* (A, B), *S. aureus* (C, D), and *P. stutzeri B27* (E, F) of Cu_2_O/P, and Cu_2_O/Ag/P particles at different diluted concentrations after 24 h of culturing at 37 °C. For agar dishes of *E. coli*, and *S. aureus*: (1) undiluted, (2) diluted 10 times, (3) diluted 20 times, (4) diluted 40 times, (5) diluted 80 times, (6) diluted 160 times, (7) Ampicillin 0,25 mg/mL, (8) RO water. For agar dishes *P. stutzeri B 27*: (1) undiluted, (2) diluted 10 times, (3) diluted 20 times, (4) diluted 40 times, (5) diluted 80 times, (6) diluted 160 times, (7) diluted 320 times (8) Ampicillin 0,25 mg/mL, (9) RO water.

Resistance to two bacterial strains *E. coli* and *S. aureus*, of Cu_2_O, Cu_2_O/Ag, Cu_2_O/P, and Cu_2_O/Ag/P particles at high to low concentrations was presented in Tables 5 and 6. It was clear that the MIC value of Cu_2_O particles for S. aureus was much lower than that of the Cu_2_O/Ag/PEG particles. Moreover, the MIC value of Cu_2_O/P was determined at lower concentrations and diluted 80 to 160 times, while the MIC value of Cu_2_O/Ag/P was determined at lower concentrations and diluted to 320 times. It demonstrated the unique antibacterial activity of Ag particles, as mentioned above after being attached to the Cu_2_O particles, which gradually increased the antibacterial activity many times. In addition, the MIC value of bacteria *P. stutzeri* B27 by Cu_2_O/P and Cu_2_O/Ag/P particles were investigated and shown in Figure 12. From the data in Tables 5 and 6, it could be seen that the two samples of Cu_2_O/P and Cu_2_O/Ag/P particles were relatively resistant to this type of bacteria with the inhibition zone in the sample not diluted having a value of 14 mm, and 20 mm, respectively. Therefore, the Cu_2_O/Ag/P particles had better antibacterial activity than the Cu_2_O/P particles, and both particle samples were resistant to all three strains of bacteria: *E. coli*, *S. aureus*, and *P. stutzeri* B27. Lastly, observing the agar dishes in Figure 12, the particle samples in positions 2, 3, 4, 5, 6, and 7 had a lower concentration than the samples in position 1 (the sample with the particle concentration had yet to be diluted). It suggests a significant reduction in the antibacterial ability of the particle samples at that location.[^34^, ^35^]

**Table 5:** MIC for different bacteria by Cu_2_O, Cu_2_O/Ag, Cu_2_O/P, Cu_2_O/Ag/P particles.

No.	Samples	Concentration (mg/mL)	Inhibition zone (D‐d) (mm)		
			*E. coli*	*S. aureus*	*P. stutzeri B27*
1	Cu_2_O/PEG undiluted	100	14±2	28±5	14±2
2	Cu_2_O/PEG diluted 10 times	10	8±2	22±1	8±1
3	Cu_2_O/PEG diluted 20 times	5	6±1	20±3	6±1
4	Cu_2_O/PEG diluted 40 times	2.5	4±0	10±2	4±0
5	Cu_2_O/PEG diluted 80 times	1.25	2±0	0	0
6	Cu_2_O/PEG diluted 160 times	0.625	0	0	0
7	Cu_2_O/PEG diluted 320 times	0.3125	0	0	0
8	Positive control (+)	10	30	53	26
9	Negative control (−)	‐	0	0	0

**Table 6:** MIC for different bacteria by Cu_2_O/Ag/P particles.

No.	Samples	Concentration (mg/mL)	Inhibition zone (D‐d) (mm)		
			*E. coli*	*S. aureus*	*P. stutzeri B27*
1	Cu_2_O/Ag/PEG undiluted	100	18±3	34±2	20±2
2	Cu_2_O/Ag/PEG diluted 10 times	10	14±1	30±4	16±2
3	Cu_2_O/Ag/PEG diluted 20 times	5	12±2	20±3	11±1
4	Cu_2_O/Ag/PEG diluted 40 times	2.5	10±2	14±2	10±2
5	Cu_2_O/Ag/PEG diluted 80 times	1.25	8±2	7±2	7±2
6	Cu_2_O/Ag/PEG diluted 160 times	0.625	6±1	5±0	2±0
7	Cu_2_O/Ag/PEG diluted 320 times	0.3125	0	0	3±0
7	Positive control (+)	10	30	53	53
8	Negative control (−)	–	0	0	0

### 2.9 Properties of emulsion acrylic coatings contained Cu_2_O, Cu_2_O/Ag particles

Table 7 shows symbols of investigated emulsion acrylic (AC) coatings containing Cu_2_O and Cu_2_O/Ag particles and their main mechanical properties, including adhesion, relative hardness, abrasion resistance, and pencil hardness. First, the data in Table 7 shows that the coatings′ physical properties of AC coatings have improved after adding Cu_2_O and Cu_2_O/Ag particles. Specifically, when comparing the neat AC coating samples with the coating contained the Cu_2_O, Cu_2_O/P or Cu_2_O/Ag, Cu_2_O/Ag/P particles, the adhesion value of AC coatings was increased from 0.72 to 0.83, 0.98, 1.01 MPa and the highest value reached 1.22 MPa for coating sample contained Cu_2_O/Ag/P particles. The relative hardness values and abrasion resistance of AC coatings were also increased in the order of samples AC < AC/Cu_2_O < AC/Cu_2_O/P < AC/Cu_2_O/Ag < AC/Cu_2_O/Ag/P. The maximum value of relative hardness and abrasion resistance of AC coatings was also obtained for AC/Cu_2_O/Ag/P coating samples with values of 240 L/mil and 100 L/mil, respectively. It was explained by the regular dispersion of Cu_2_O/Ag/P particles into the AC matrix, which could allow the formation of a uniform structure and act as a role of barrier that can prevent the impact of external force on the coating.[^36^, ^37^, ^38^, ^39^] The pencil hardness of coating samples was affected negligibly in the presence of microparticles.

**Table 7:** Mechanical properties of emulsion acrylic coatings contained Cu_2_O, and Cu_2_O/Ag particles.

Samples	Adhesion force (MPa)	Relative hardness	Abrasion resistance (L/mil)	Pencil hardness
AC	0.72±0.05	125±6.41	68±0.83	5H
AC/Cu_2_O	0.83±0.06	180±3.73	75±1.41	5H
AC/Cu_2_O/P	0.98±0.18	189±7.42	85.7±1.92	5H
AC/Cu_2_O/Ag	1.01±0.08	209±7.27	89.2±2.55	4H
AC/Cu_2_O/Ag/P	1.22±0.09	240±8.81	100±2.62	5H

### 2.10 Morphology of emulsion acrylic coatings contained Cu_2_O, Cu_2_O/Ag particles

**Figure 13:**
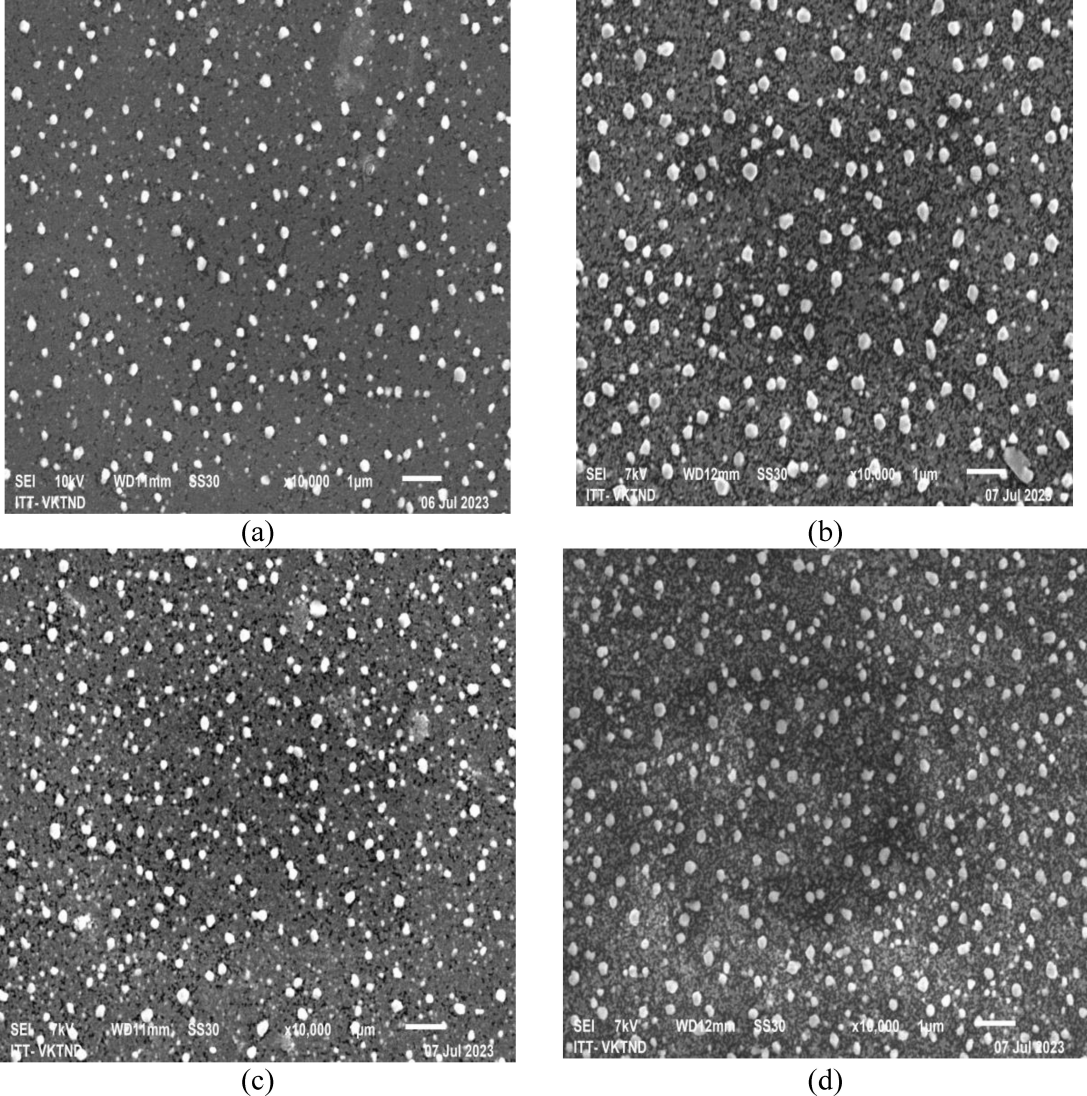
Images SEM of AC coatings (a): AC/Cu_2_O, (b): AC/Cu_2_O/Ag, (c): AC/Cu_2_O/P, (d): AC/Cu_2_O/Ag/P.

The SEM images of emulsion AC coatings containing Cu_2_O, Cu_2_O/Ag, Cu_2_O/P, and Cu_2_O/Ag/P particles are performed in Figure 13. When comparing SEM images A and C (corresponding to the AC coatings containing Cu_2_O and Cu_2_O/P particles, respectively), it was clear that the Cu_2_O/P particles were dispersed more regularly into the AC matrix. Their particle size was substantially smaller, and the particle density was higher. Similarly, the Cu_2_O/Ag/P particles were also regularly dispersed well in AC coatings compared to Cu_2_O/Ag thanks to the presence of PEG surfactant in the synthesis process of Cu_2_O and Cu_2_O/Ag particles.

### 2.11 Antibacterial activity of emulsion AC coatings contained Cu_2_O, Cu_2_O/Ag particles

The antibacterial activity of AC coatings was also evaluated by determining their inhibition zone. The results in Table 8 showed that the AC coating contained Cu_2_O, Cu_2_O/Ag particles were resistant to two strains of bacteria, *E. coli* and *S. aureus*. This is thanks to the diffusion of Cu_2_O and Cu_2_O/Ag particles from the AC‐based coatings, which inhibits the growth of bacteria. The AC/Cu_2_O and AC/Cu_2_O/P coating can inhibit the growth of bacteria with an inhibition zone from 3–6 mm. The AC/Cu_2_O/P coating exhibits a better antibacterial ability than the AC/Cu_2_O coating because the Cu_2_O/P can inhibit the growth of bacteria better than the Cu_2_O, as aforementioned.

**Table 8:** Inhibition zone (D‐d) of Cu_2_O/AC, Cu_2_O/P/AC, Cu_2_O/Ag/AC, and Cu_2_O/Ag/P/AC coatings.

No.	Name of sample	Inhibition zone (D‐d) (mm)	
		*E. coli*	*S. aureus*
1	AC/Cu_2_O	3±0	3±0
2	AC/Cu_2_O/P	6±0	5±0
3	AC/Cu_2_O/Ag	10±1	8±1
4	AC/Cu_2_O/Ag/P	14±1	12±1
5	AC	0	0
6	Negative control	0	0
7	Ampicillin	22	34

Interestingly, the AC/Cu_2_O/Ag and AC/Cu_2_O/Ag/P can inhibit the growth of two bacterial strains more strongly, with an inhibition zone from 10–14 mm for *E. coli* and 8–12 mm for *S. aureus*. This is evidence for the synergetic effect of Cu_2_O and AgNPs in bactericidal properties.[^5^, ^38^, ^40^, ^41^] The particle size of Cu_2_O/Ag/P is smaller than that of Cu_2_O/Ag; they can disperse better in AC coating. Therefore, the antibacterial activity of AC/Cu_2_O/Ag/P coating is better than AC/Cu_2_O/Ag coating.

## 3 Conclusions

This report developed a simple method for fabricating Cu_2_O oxide copper (I) particles and Cu_2_O/Ag particles with and without using co‐surfactants of SDS and PEG. The presence of co‐surfactants in the synthesis process of Cu_2_O and Cu_2_O/Ag particles showed an extremely positive effect on the properties of Cu_2_O and Cu_2_O/Ag particles, including particle size, contact angle, morphology, composition and bactericidal activity. The Cu_2_O/P and Cu_2_O/Ag/P particle samples had the smallest particle size value (200–400 nm) and superior antibacterial ability (32 mm for *E. coli* and 36 mm for *S. aureus*) compared to the other samples. A heterogenous structure of Cu_2_O/Ag was confirmed through UV‐Vis, EDS mapping and TEM analysis. Moreover, the Ag NPs was also formed on the surface of Cu_2_O/Ag samples. The Cu_2_O/P had antibacterial ability at extremely low dilutions 80 times, while the Cu_2_O/Ag/P sample was even better when diluted 160 times. The most memorable thing was that these particle samples inhibit the growth of two common bacteria strains, *E. coli*, *S. aureus*, and can also kill *P. stutzeri* B27 marine bacteria. It was meaningful when applied in paint manufacturing to prevent barnacles for ships and boats at sea. Finally, the particle samples were applied to manufacture AC emulsion coatings. They showed excellent results when the mechanical properties of the coatings were highly stable and when coatings were created with very high bacterial ability.

## Author Contributions

DXT: Writing‐original draft, investigation. THN: Investigation, Formal analysis. NTC: Methodology, Supervisor, Formal analysis, Validation. NTBM: Investigation. TH: Conceptualization, Funding acquisition, Writing‐Reviewing and Editing.

## Conflict of interests

The authors declare no conflict of interest.

4

## Data Availability

The data used to support the findings of this study are included within the article.
